# Understanding the Functions of Long Non-Coding RNAs through Their Higher-Order Structures

**DOI:** 10.3390/ijms17050702

**Published:** 2016-05-17

**Authors:** Rui Li, Hongliang Zhu, Yunbo Luo

**Affiliations:** Department of Food Biotechnology, College of Food Science and Nutritional Engineering, China Agricultural University, Beijing 100083, China; spxylirui@cau.edu.cn (R.L.); lyb@cau.edu.cn (Y.L.)

**Keywords:** long non-coding RNA, molecular mechanisms, structure, function, technologies

## Abstract

Although thousands of long non-coding RNAs (lncRNAs) have been discovered in eukaryotes, very few molecular mechanisms have been characterized due to an insufficient understanding of lncRNA structure. Therefore, investigations of lncRNA structure and subsequent elucidation of the regulatory mechanisms are urgently needed. However, since lncRNA are high molecular weight molecules, which makes their crystallization difficult, obtaining information about their structure is extremely challenging, and the structures of only several lncRNAs have been determined so far. Here, we review the structure–function relationships of the widely studied lncRNAs found in the animal and plant kingdoms, focusing on the principles and applications of both *in vitro* and *in vivo* technologies for the study of RNA structures, including dimethyl sulfate-sequencing (DMS-seq), selective 2′-hydroxyl acylation analyzed by primer extension-sequencing (SHAPE-seq), parallel analysis of RNA structure (PARS), and fragmentation sequencing (FragSeq). The aim of this review is to provide a better understanding of lncRNA biological functions by studying them at the structural level.

## 1. Introduction

Two types of RNA molecules exist [1]: messenger RNA (mRNA) molecules, which possess the ability to encode the amino acid sequence of proteins, and non-coding RNAs (ncRNAs), which lack or have very little protein-coding potential [2]. mRNAs, an essential component of the central dogma of molecular biology, are known for their crucial roles as intermediaries conveying genetic information from DNA to the ribosomes and mediating protein synthesis [3]. With the rapid development and application of high-throughput deep sequencing, it was shown that although ~90% of the eukaryotic genomeis transcribed, mRNAs account only for 1%–2% of total RNAs, suggesting that a large number of RNA molecules are ncRNAs [4]. NcRNAs can be further classified as “housekeeping” ncRNAs and “regulatory” ncRNAs, based on their functions [5]. The former includes ribosomal RNA (rRNA), transfer RNA (tRNA), small nuclear RNA (snRNA), and small nucleolar RNA (snoRNA), while the latter usually refers to small ncRNA (sncRNA) and long non-coding RNA (lncRNA) [5]. SncRNAs have been the focus of molecular biology research over the last decade, and it was demonstrated that they are involved in the regulation of their target genes at both transcriptional and post-transcriptional levels [6]. However, lncRNA investigations have begun only recently.

**Figure 1:**
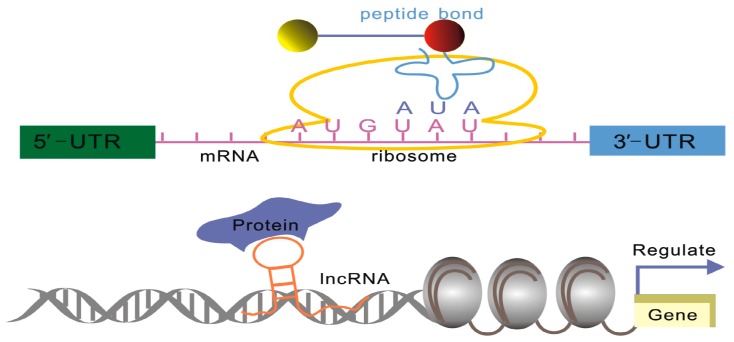
Differences in the structure and sequence between mRNA and lncRNA. The mRNA primary coding sequence (CDS) plays a significant role in the translation, while lncRNAs regulate target gene expression through the interactions between their higher-order structures and major partner proteins.

It is generally believed that lncRNAs, RNA molecules longer than 200 nucleotides, belong to a group of RNAs with broad biogenesis, and that these molecules are always capped and polyadenylated [7]. Initially, lncRNAs were considered “transcriptional noise” without any biological function. However, thousands of reports in recent years have demonstrated that lncRNAs, which interact with DNA, RNA molecules, and transcription factors, participate in various biological processes, such as DNA methylation, histone modification, and chromatin remodeling, resulting in the downregulation or overexpression of target genes [8]. There are four main ways in which all lncRNAs execute their functions: as signals, decoys, guides, or scaffolds [9]. Additionally, lncRNAs are often characterized by their tissue- and time-specific developmental expression patterns [5,8,10]. With the discovery of many biological functions of lncRNAs, their higher order structures have received an increasing amount of attention. Different studies have reported that the primary sequences of lncRNAs, unlike mRNA structural features,show very little conservation, but their secondary and tertiary structures are highly conserved and might be potentially related to their biological functions (Figure 1) [10,11]. For example, SRA (steroid receptor RNA activator), a breast cancer-linked lncRNA, which co-activates several nuclear receptors and proteins, is reported to have highly conserved helices, terminal loops, and bulges in many species [12]. Four lncRNAs designated TalncRNA18, TalncRNA73, TalncRNA106, and TalncRNA108, which are associated with the response to stripe rust pathogen stress in wheat, were shown to have the same stem structures [13]. In addition to lncRNA structures related to the biotic stress response, highly conserved domains of lncRNAs associated with the abiotic stress response were also found. Furthermore, lncRNAs responding to salt stress often have UUC motifs, while lncRNAsmediating the response to cold contain AU-rich stem-loop structures [14].

Initially, methods such as nuclear magnetic resonance (NMR) and X-ray crystallography were used for the investigations of RNA structures [15]. However, since RNA molecules have high degeneration rates and are difficult to crystallize, these methods cannot accurately identify RNA functional regions. Currently, researchers mainly use chemical and enzymatic strategies to study highly conserved structures of lncRNAs [16]. The rapid development of lncRNA structure probing methods helps researchers gain a deeper understanding of lncRNA structure-function relationships. In this short review, we will focus on the relationships between lncRNA structures and their functions. Furthermore, some tools widely used for the investigations of highly ordered RNA structures will be systematically discussed as well, and the indications for future development will be given.

## 2. lncRNA Structure and Biological Function Relationships

lncRNAs, which are frequently involved in transcriptional, post-transcriptional, and epigenetic processes, are currently the focus of genetic research [8,17]. Previous studies have shown that the secondary and tertiary structures of lncRNAs are highly conserved and that these highly conserved structures are strongly related to lncRNA biological functions [11,18]. Although thousands of lncRNAs have been discovered in recent years, many of their functional sites remain unknown [19]. In the following sections, we will discuss the structure–function relationships of lncRNAs found in animals and plants that have been extensively studied.

### 2.1. lncRNAs in Animals

#### 2.1.1. Xist: Repetitive Elements Involved in Protein Complex Recruitment

During the early stages of embryonic development, genes on the X chromosomes in female mammals are inactivated in order to achieve the same expression levels of X-chromosomal genes in male mammals [20,21]. This widely spread phenomenon is called X-chromosome inactivation (XCI), and the regulatory genes involved in XCI are located at the X-inactivation center [22]. Among these genes, the *Xist* (X-inactive specific transcript) gene plays an essential role in XCI. The lncRNA Xist, 17 kb in length, is a transcript of *Xist*, which initiates XCI by coating the X chromosome in order to regulate cis X inactivation (Xi), and by recruiting modifying complexes, such as polycomb repressive 2 (PRC2), to specific sites on Xi, resulting in histone H3 lysine K27 trimethylation (H3K27me3) and X-linked gene silencing [21,23]. Another lncRNA involved in this process, termed Tsix, is an antisense transcript of *Xist*, which has the opposite effect and can prevent Xist from coating the X chromosome [24,25]. Maenner *et al.* found a repeated element in Xist, which contains eight repeats, termed A-repeat; this region represents the most conserved Xist region [26]. Its 2D structure shows two long stem-loop structures in the A-repeat, and each stem-loop contains four repeats, which were shown to be associated with PRC2 recruitment [26]. It was demonstrated that several segments of the A-repeat assist with the recruitment of particular PRC2 components, but also that the increase in the efficacy of binding to the entire complex was observed when the entire A-repeat was involved, suggesting that the A-repeat plays a significant role in XCI by regulating the rate of PRC2 recruitment [26]. Additionally, a novel, highly stable tetraloop motif, the AUCG loop, was found in the 5’ region of the human A-repeat; the integrity of this structure was closely related to Xist silencing [27]. It was reported that the 3’ region of the A-repeat plays a significant role in intermolecular duplex formation and that any mutations that disrupt the structure of this region, as observed *in vitro*, can compromise the biological functions of the A-repeat *in vivo* [27].

**Figure 2:**
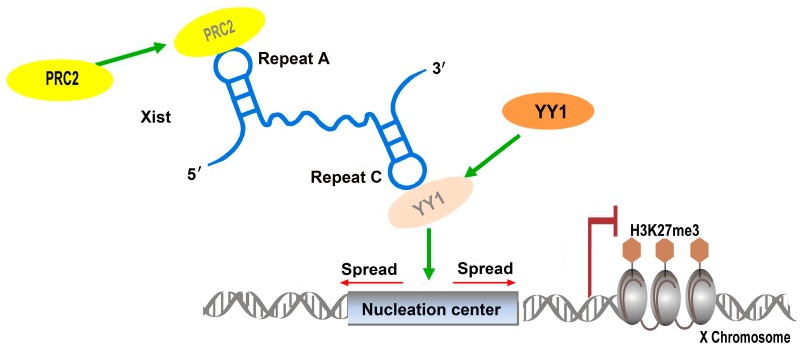
Xist repetitive element functions during X-chromosome inactivation. A-repeat, which contains two long stem-loop structures, is involved in PRC2 binding, while C-repeat binds YY1, assisting Xist-PRC2 complex in targeting the specific sites on Xi, and inducing histone H3 lysine K27 trimethylation (H3K27me3) and X-linked gene silencing.

In addition to the A-repeat, a C-repeat, which binds YY1 transcription factor and contains four recurring hairpins, was found to be involved in the localization and tethering of the Xist–PRC2 complex to the specific sites of X chromosome, inducing X-linked gene silencing (Figure 2) [28]. Although C-repeat structure probing showed only a moderate rate of conservation between different species, a 441-nucleotide subdomain containing 55 nucleotides downstream of the last C-repeat is highly structured and conserved in many species [29]. The disruption of this subdomain leads to Xist dissociation from Xi, indicating the importance of this conserved structure for Xist functions [29].

Recently, Lv *et al.* confirmed the significance of Xist D-repeat in XCI using CRISPR/Cas9 (clustered regularly interspaced short palindromic repeats (CRISPR)-associated endonuclease 9) [30]. The D-repeat knockout directly led to a significant decrease of *Xist* levels, leading to the upregulation of X-linked genes [30]. The abundance and wide distribution of repetitive elements in lncRNAs suggest that they may play significant roles in exerting biological functions of lncRNA.

#### 2.1.2. RoX: Tandem Stem-Loops Direct MSL Complex Assembly

Another widely discussed dosage compensation effect regulated by an lncRNA is X-chromosome dosage compensation in *Drosophila*. Unlike the previously discussed X-chromosome inactivation dosage compensation, genes on the single X chromosome in *Drosophila* males must be upregulated in order to match the expression levels of the genes on the two X chromosomes in females [31]. Initial research revealed that this upregulation is mediated by male-specific lethal (MSL) complex, which includes two lncRNAs (roX1 and roX2) and five proteins (MSL1, MSL2, MSL3, MOF (males absent on the first), and MLE (maleless)) [32,33]. This complex is able to bind to the high-affinity sites (HAS) on X-chromosome and direct histone H4 lysine16 acetylation (H4K16ac), while two lncRNAs involved in the formation of this complex, RNA on the X1 (roX1) and RNA on the X2 (roX2), serve as scaffolds essential for X-chromosome targeting [33].

**Figure 3:**
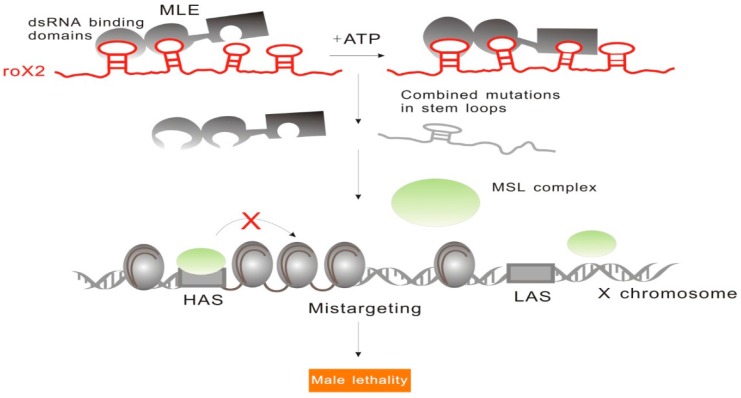
RoX2 tandem stem-loops are involved in MSL complex assembly. RoX2 tandem stem-loops are highly conserved. MLE binding to the different parts of tandem stem-loops has different ATP requirements. MLE binding to the first half of roX2 does not require ATP, while binding to the second half is ATP-dependent. Only when combinatorial mutations occur in stem loops, roX2 is no longer able to recruit MSL, which results in the loss of dosage compensation and male lethality.

In order to unveil the specific mechanisms underlying MSL interactions with roX1 and roX2, Ilik *et al.*, who suggested that MLE (maleless) RNA helicase and MSL2 (male-specific lethal 2 homolog) ubiquitin ligase are required for the association of roX lncRNAs with the complex, showed that the tandem stem-loop structures in roX1 (D1–D3) and roX2 exon3 were involved in the interactions with MLE and MSL2 [34]. RoX1 D3 region showed the highest MLE-binding capacity, and the binding of MLE to different domains of roX2 showed different ATP requirements. This complex is able to bind to the first half of roX2 in an ATP-independent manner, while the binding to the second half of this molecule is ATP-dependent [34]. Additionally, only when the combinatorial mutations occurred in tandem stem-loops of roX2, loss of dosage compensation occurred as well, indicating the existence of structural redundancy in lncRNAs (Figure 3). These results show that the functions of roX during the recruitment of MSL complex assemblies are determined by the specific tandem stem-loop domains.

#### 2.1.3. minHOTAIR Binds PRC2, while D4 Domain Recruits the LSD1 Complex

HOTAIR (HOX antisense intergenic RNA), which contains 2158 nucleotides, is an antisense transcript of *HOXC* [35]. It is a trans-acting factor that regulates *HOXD* gene expression by recruiting PRC2 and lysine-specific demethylase 1 (LSD1) to the specific sites [36]. The PRC2 complex is comprised of three core protein subunits, EZH2, EED, and SUZ12, which are involved in the regulation of H3K27me3, while LSD1 leads to the demethylation of histone H3 lysine 4, which is crucial for transcriptional activation [37]; its overexpression may lead to tumorigenesis [38,39].

Sophisticated biological functions are often determined by highly conserved structures, and this is the case with HOTAIR as well. More than 50% of HOTAIR nucleotides are base-paired and this highly structured lncRNA contains 56 helical segments, 38 terminal loops, 34 internal loops, and 19 junction regions [40]. Previous studies showed that the 300-mer domain at the 5’ terminus of HOTAIR is involved in PRC2 binding [37]. However, a much shorter section was determined by Wu *et al.* to contain the minimal binding motif of HOTAIR (minHOTAIR), and its 2D structure was established by nuclease digestion experiments [41]. An 89-mer domain at the 5’ end of HOTAIR, termed minHOTAIR, includes two duplex regions connected by a 10-nucleotide single strand (ss) RNA linker. The disruption of this highly conserved structure affects PRC2 binding to HOTAIR, which demonstrates a close relationship between lncRNA biological functions and structural conservation [41].

**Figure 4:**
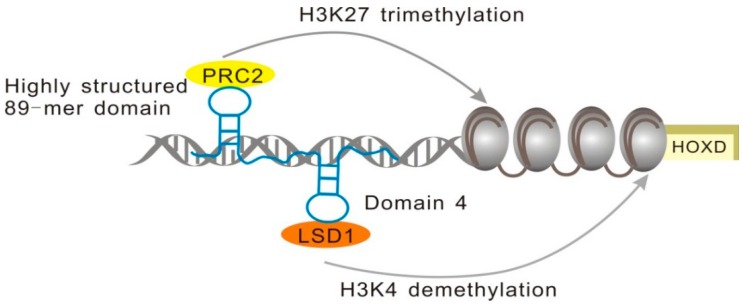
minHOTAIR and D4 regions of HOTAIR recruit PRC2 and LSD1, respectively, in order to regulate *HOXD* expression.

In contrast, the LSD1 complex is recruited using the motif on the 3’ end of HOTAIR [37]. This motif is a 646-mer domain very different from PRC2 recruitment domain, and nucleotides between the positions 1500 and 2148 contribute to the formation of this functional domain [37]. Somarowthu *et al.* determined that the nucleotide sequence involved in the LSD1 complex binding motif is very similar to the sequence of a conserved domain, D4, which contains 20 helices, 13 terminal loops, and seven junctions (Figure 4) [42]. Their findings show that the functions of HOTAIR in the recruitment of different histone modification complexes are achieved mainly by the intricate and modular nature of its secondary structures.

#### 2.1.4. MALAT1: Triple Helix Structure Explains the High Stability of Long Nuclear-Retained Transcripts

MALAT1 (metastasis associated lung adenocarcinoma transcript 1), also called NEAT2 (nuclear enriched abundant transcript 2), is a type of long nuclear-retained transcript that was shown to be associated with cancer cell metastases. It is widely expressed in both human and mouse tissues, and it is overexpressed in many human carcinomas [43,44]. Aberrant expression of MALAT1 leads to a decrease in patient survival [45]. This lncRNA is able to regulate alternative splicing by modulating the cellular levels of serine/arginine (SR) factors [46].

**Figure 5:**
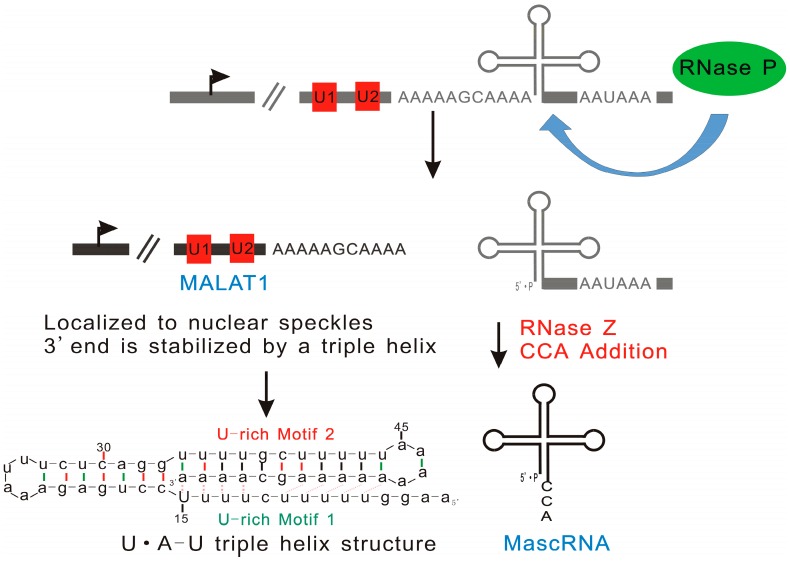
Triple helix structure of MALAT1 explains its high stability. RNase P is involved in the generation of the 3’ end of MALAT1 and the 5’ end of tRNA-like cytoplasmic RNA designated as mascRNA. A triple helix (U•A-U) formed by the conserved poly(A)- and its flanking U-rich motifs prevents the degradation of MALAT1 by exonucleases.

Unlike the 3’ or 5’ ends of other RNAs that are produced by canonical cleavage, RNase P is responsible for the generation of the 3’ end of MALAT1 and the 5’ end of tRNA-like cytoplasmic RNA designated as MALAT1-associated small cytoplasmic RNA (mascRNA) [47]. Wilusz *et al.* investigated the structure of MALAT1 and other nuclear-retained transcripts, and they suggested that the short poly(A)-rich tract at the 3’ ends of these transcripts may exist in all long nuclear-retained transcripts [48]. Considering that the poly(A) tail of mRNA increases its stability, and the long half-life of MALAT1, it has been suggested that the short poly(A) tail-like moieties may correlate with the stability of MALAT1 and its resistance to exonucleases [49]. A recently published study performed by this group showed that the highly conserved poly(A)- and its neighboring U-rich motifs act together in order to protect the 3’ end of MALAT1 from the activity of exonucleases through base pairing [48]. However, it was found that base pairing between U-rich motif 2 and poly(A)-rich tract only partially contributes to MALAT1 stability. Further analysis revealed that a triple helix U•A-U (where • and -represent Hoogsteen and Watson-Crick faces, respectively), formed by U-rich motif 1 interacting with A-U duplex through Hoogsteen hydrogen bonding, is involved in the maintenance of the transcript stability (Figure 5) [49,50]. A similar triple helix structure has been found in multiple endocrine neoplasia-β (MENβ) RNA, which is another lncRNA with nuclear localization and a long half-life [50]. Therefore, it appears that the formation of the triple helixes on 3’ ends is a common way for long nuclear-retained transcripts to avoid exonuclease degradation, which enhances their biological functions.

#### 2.1.5. Gas5 Acts as a Decoy for the Glucocorticoid Receptor through Structure Transformation

**Figure 6:**
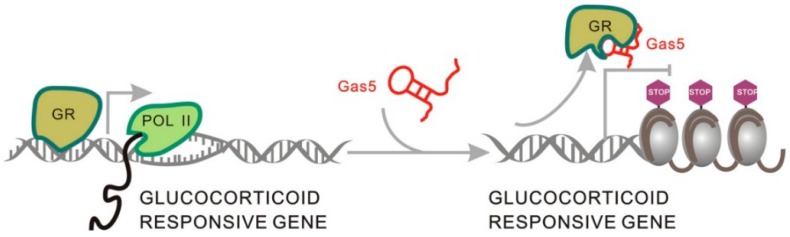
The role of Gas5 secondary structure transformation in glucocorticoid signal transduction. Gas5 serves as a decoy for GR and removes it from the signaling pathway by changing its secondary structure. POL: Polymerase.

Growth arrest-specific transcript 5 (Gas5) was shown to be downregulated in many cancer tissues, and therefore it has long been considered a cancer-related lncRNA [51]. Recently, Kino *et al.* showed that it also acts as a decoy for glucocorticoid receptor (GR), regulating target gene expression [52]. When Gas5 is not present in the glucocorticoid signaling pathway, glucocorticoid (GC) first binds to GR in cytoplasm, forming a GC–GR complex, which is transported into the nucleus, where it binds glucocorticoid response elements (GREs) via its DNA binding domains, leading to the activation of gene expression [11].Gas5 is able to mimic GREs through changes in its secondary structure and competitively binds to GR, effectively blocking glucocorticoid signal transduction by removing GR molecules from the signaling pathway (Figure 6) [52]. By comparing human and mouse Gas5 structures, researchers found that even though the nucleotide sequences of Gas5 transcripts are not highly homologous, the functional motif able to bind GR is conserved across species [52]. Therefore, it was suggested that the mechanism of Gas5 interactions with the transcription factor through a structural transformation may exist in other lncRNAs with similar domains, but this requires further validation [18,53].

### 2.2. lncRNAs in Plants

Even though, compared with lncRNA studies in animals, fewer lncRNAs have been functionally characterized in plants [54], a number of lncRNAs that participate in plant reproductive development, pathogen stress responses, transcriptional gene silencing, male sterility, and cell differentiation have been identified in recent years [55,56,57], and their functional domains have been determined as well.

#### 2.2.1. IPS1 Functions as an Endogenous Target Mimic Using Its 23-Nucleotide Conserved Motif

**Figure 7:**
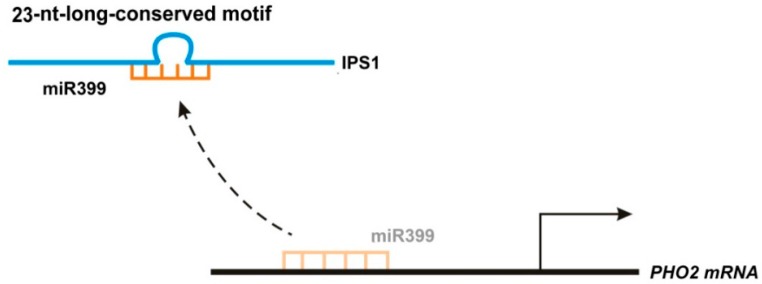
IPS1 functions as an endogenous target mimic through a 23-nucleotide (nt)-long conserved motif. The conserved 23-nt-long motif of IPS1, which shows imperfect complementarity with miR399, ensures binding with miR399. This leads to an increased expression of miR399 target genes and changes in phosphate content, since miR399 can no longer affect its targets.

*Arabidopsis thaliana* has long been a model species for studies of lncRNA functions in plants. An lncRNA named Induced by Phosphate Starvation 1 (IPS1) was found to be associated with shoot phosphate (Pi) content [58,59]. Phosphate starvation-induced miR399 reduces *PHO2* mRNA accumulation [58], but IPS1, which regulates *PHO2* through a mechanism called endogenous target mimicry (eTM), serves as a decoy for miR399 in phosphate-starved plants [58]. The conserved 23-nucleotide (nt)-long motif of IPS1, which shows imperfect complementarity with miR399, mainly ensures IPS1 and miR399 binding, while its 3-nt central mismatch loop at the expected miRNA cleavage site enables secure binding of miR399, ensuring that miR399 can no longer affect its target, which results in increased expression of target genes and changes in phosphate content (Figure 7) [58]. The target mimic region of IPS1 is highly conserved in many plant species. It was suggested that eTM exists in both plant and animal species and the identification of short conserved motifs in lncRNAs would provide new insights into lncRNA–microRNA interaction mechanisms [60].

#### 2.2.2. Functional Domains of COOLAIR and COLDAIR Are Involved in the Repression of *Flowering Locus C (FLC)*

**Figure 8:**
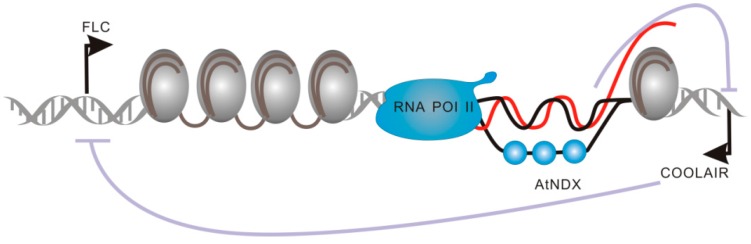
R-loop structures covering the COOLAIR promoter repress COOLAIR transcription. *FLC*: *Flowering Locus C*.

Flowering transition is a crucial step for plant reproductive development, and *FLC* has long been known as a regulator of flowering in plants [61]. Recently, the studies showed that two vernalization-induced lncRNAs, COOLAIR (Cold Induced Long Antisense Intergenic noncoding RNA) and COLDAIR (Cold Assisted Intronic noncoding RNA), could regulate *A. thaliana* flowering time through *FLC* repression [62]. COOLAIR, transcribed from the 3’ end of *FLC*, represents a group of long non-coding antisense RNAs [62,63]. Even though it is not indispensable for the direct epigenetic silencing of *FLC*, it significantly promotes *FLC* transcriptional repression [64]. Recently, COOLAIR transcription was found to be correlated with the R-loop structure, formed by an RNA–DNA hybrid, together with a displaced ssDNA strand [65]. R-loops were initially considered transcriptional byproducts without any biological functions. However, Sun *et al.* showed that the R-loop, covering the COOLAIR promoter, is able to promote *FLC* expression by repressing COOLAIR transcription (Figure 8) [65]. The R-loop structure has been shown to have multiple roles, and these structures may play crucial roles in the regulation of gene expression in many organisms.

COLDAIR, originating from the first intron of *FLC*, has the characteristics of transcripts transcribed by Pol IV and Pol V, including 5’ capped structure, but no poly(A) tail [66]. The knockdown of COLDAIR by RNA interference (RNAi) compromises the vernalization response, indicating its role in *FLC* epigenetic silencing [5]. It acts in the same way as Xist and HOTAIR, which serve as scaffolds for the recruitment of PRC2 complexes to specific loci and induce epigenetic silencing [5]. This indicates that the epigenetic silencing mediated by PRC2 recruitment through lncRNAs is an evolutionarily conserved mechanism in both animals and plants [67]. Recent studies show that the double stem-and-loop structures formed by fewer than 100 nts in lncRNAs are involved in PRC2 recruitment *in vitro*, demonstrating the significance of lncRNA structures for the determination of their functional roles [68].

#### 2.2.3. LDMAR: lncRNA Structural Integrity Is Required in Order to Exert Biological Functions

Photoperiod is known to be very important in the regulation of plant growth and development. Recently Ding *et al.* found that a 1236-nt long lncRNA, termed long-day-specific male-fertility-associated RNA (LDMAR), plays a significant role in the regulation of photoperiod-sensitive male sterility (PSMS) in rice Nongken 58S (NK 58S), a spontaneous mutant of Nongken 58N (NK 58N) [69]. Under long-day conditions, the reproductive development of both NK 58S and NK 58N requires a high expression of LDMAR. Several studies showed that the methylation level of LDMAR promoter regions in NK 58S was considerably higher than the level in NK 58N, leading to a much lower LDMAR expression in NK 58S, and finally resulting in PSMS [69]. Further analyses showed that this phenomenon was directly caused by LDMAR structural changes. Compared with the structure of LDMAR in NK 58N, the secondary structure of LDMAR in NK 58S was altered by spontaneous mutations, generating several small RNAs, which are involved in an RNA-dependent DNA methylation (RdDM) pathway, thereby increasing the methylation in the promoter region of LDMAR [70]. Therefore, it was shown that the transcription level of LDMAR is reduced under long-day conditions and PSMS appears because of the decrease in LDMAR levels [71]. Although the specific structure associated with LDMAR expression and the underlying biochemical mechanisms remain unknown, LDMAR functional studies showed that structural integrity is crucial for lncRNA biological function.

#### 2.2.4. ENOD40 Highly Structured Motif Is Involved in MtRBP1 Binding and Trafficking

The *ENOD40* (*early nodulin 40*) gene was initially found to play a significant role in the root nodule organogenesis of leguminous plants [72,73]. It was also suggested that *ENOD40* participates in other non-symbiotic plant developmental processes, including the differentiation of vascular bundles [73]. The abundance and degree of conservation of *ENOD40* in plants suggest that this gene may have conserved biological functions. Its transcript *ENOD40* RNA, which contains a short open reading frame mRNA (sORF-mRNA) was shown to have a bi-functional role in the process of nodule organogenesis [72,74]. Rohrig *et al.* found that the conserved domains at the 5’ end of ENOD40 in soybeans encode for two 12- and 24-amino acid peptides *in vitro* [75]. Both of these peptides are able to affect sucrose synthase activity by binding to a component of sucrose synthase named nodulin 100, following its translation [75].

Comparing ENOD40 structure in different leguminous species, Girard *et al.* showed that five domains in ENOD40 were highly conserved, and that uridine residues were numerous in most of these conserved terminals and loops [76]. However, ENOD40 is not restricted to symbiotic plant development [73], and new studies have shown that it can function as a guide, directing the relocation of NSR (nuclear speckle RNA-binding proteins). A novel NSR, MtRBP1 (*Medicago truncatula* RNA Binding Protein 1), can be transported by ENOD40 into cytoplasmic granules during nodulation. Mutations that impair the translation of the two peptides do not influence the trafficking activity of ENOD40, suggesting that ENOD40 has different functional roles, supported by different motifs [77]. Though *ENOD40* functions as both a protein-coding and non-coding gene, the highly conserved RNA structures imply that ENOD40 belongs to the group of lncRNAs [72]. Furthermore, it has recently been reported that some ncRNAs have the potential to encode small peptides as well, indicating that ENOD40 should be categorized as an lncRNA [78]. Later in *A. thaliana*, Bardou *et al.* found a similar lncRNA-ASCO-RNA (Alternative Splicing Competitor RNA), previously named lnc351, that could modulate alternative splicing through binding with NSR *in vivo* [79]. Although structures of ASCO for NSR binding have not been revealed yet, we could infer that ASCO might also be highly structured. ENOD40 studies show that highly structured lncRNAs can simultaneously determine multiple biological functions.

## 3. Technologies Used in the Structural Studies of RNAs

There is no doubt that tools used for the investigation of RNA structures significantly contribute to a rapid increase in our understanding of RNA function. Currently, the technologies for the structural characterization of RNAs encompass *in vitro* and *in vivo* methods [16]. *In vitro* methods mainly use different RNases to digest the RNA molecules of interests, while chemical reagents with cell penetration abilities are often applied for *in vivo* RNA structure probing [80]. In the following sections, we will discuss the basic principles and applications of these technologies that could potentially be applied to investigate lncRNA structures, together with the description of several lncRNA purification methods for motif determination.

### 3.1. Methodologies of lncRNA Purification for Motif Determination

**Figure 9:**
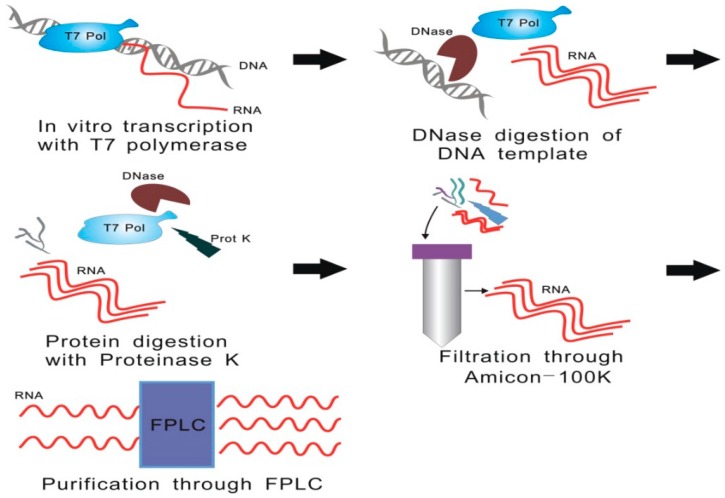
Enzymatic synthesis and purification of lncRNA. T7 RNA polymerase system is used for RNA synthesis, followed by the addition of DNase enzyme for the digestion of DNA template, and by the addition of proteinase K, which is responsible for the proteolysis of enzymes. The desired RNA is obtained by ultrafiltration and purified using size-exclusion chromatography. FPLC: Fast Protein Liquid Chromatography.

lncRNA structural or biochemical studies often require pure and homogeneous samples [81]. Therefore, lncRNA purification methods, which directly determine the quality of downstream analysis, are important for structure probing [81]. Initially, RNA purification protocols use denaturing polyacrylamide gel electrophoresis to achieve target RNA *in vitro* isolation. However, the application of these methods is limited, since denatured RNAs are often misfolded. Additionally, lncRNAs, unlike mRNAs, show little structural constraint and often form alternative conformations *in vivo*, making them even harder to analyze [82,83]. Therefore, several different approaches that avoid RNA denaturation have been developed to overcome these issues in recent years. Most of those approaches utilize affinity tag, which is involved in the immobilization of the target RNAs, and ribozyme, to elute them specifically [82]. Although this has been successfully applied for the investigation of guanine riboswitch structure, the idiosyncrasy of these methods hinders their further application. Batey and Kieft increased the applicability and reliability of this method through the introduction of MS2 coat protein for the immobilization and glm S ribozyme for the target RNA elution [82]. Subsequently, Chillón *et al.* introduced a more convenient and robust approach for lncRNA purification. Compared with the previously described approaches, this method, which does not involve RNA denaturation and affinity tag design, not only preserves lncRNA functional elements but also simplifies cloning design [84]. This newly published lncRNA protocol includes the following steps [84]: T7 RNA polymerase system is used for RNA synthesis, followed by the addition of DNase enzyme, for the digestion of DNA template, and by the addition of proteinase K, which is responsible for the proteolysis of enzymes. The desired RNA is obtained by ultrafiltration and purified using size-exclusion chromatography (Figure 9).

### 3.2. Methodologies of RNA Structure Probing in Vitro

#### 3.2.1. SHAPE-seq, SHAPE-MAP, and RING-Map

Among a number of methods for the investigation of RNA structure–function relationships *in vitro*, selective 2’-hydroxyl acylation analyzed by primer extension (SHAPE) is one of the most commonly used technologies [85].It is based on the properties of the 2’-hydroxyl group, which represents a universal chemical feature of every RNA molecule. Acetylated 2’-hydroxyl group content of RNA can be assessed in different chemical environments. Target RNA is treated with SHAPE reagents, 1-methyl-7-nitroisatoic anhydride (1M7) and *N*-methylisatoic anhydride (NMIA), which block reverse transcription and 2’-*O*-adduct formation; afterward, RNA is reverse-transcribed to cDNA. Additionally, RNA structural information is obtained by capillary/gel electrophoresis and bioinformatic analyses [86]. However, this type of the analysis of SHAPE chemical probing data can be used for the investigation of a limited number of RNAs at a time, which severely prevents its development. Lucks *et al.* improved this method by combining SHAPE probing with next generation sequencing (NGS) (SHAPE-seq) (Table 1), increasing the range of its applications, and making genome-wide RNA structure probing possible [87].

Another recently reported similar method is SHAPE-mutational profiling (SHAPE-MAP) (Table 1) [88]. Compared with SHAPE-seq, SHAPE-MAP does not involve RNA ligation and library preparation, which is time-consuming. The accuracy and reproducibility of this newly developed method have been validated through the examination of well-characterized RNA [88]. Additionally, SHAPE-MAP has allowed many improvements in HIV-1 RNA structure modeling, including the improvement of energy and pseudoknots models [88]. Mutational profiling analysis has been employed in other *in vitro* RNA structure probing techniques, including RNA interaction groups by mutational profiling (RING-MaP) (Table 1), developed by Homan *et al.*, which has been successfully used to establish the 3D RNA structure of thiamine pyrophosphate (TPP) riboswitch, P456 group I intron domain, and RNase P domain [89]. Although SHAPE chemical probing methods and RING-Map show high accuracy in RNA structure analysis, they can only be used for single-strand region analyses [90]. Dimethyl sulfate (DMS) reagent, used in RING-Map, can only modify cytosine and adenosine nucleotides, which may lead to biased results [90]; therefore the improvement of these technologies is necessary in order to increase the range of applications and accuracy.

#### 3.2.2. PARS and FragSeq

Parallel analysis of RNA structure (PARS), developed by Kertesz *et al.*, is a novel strategy for genome-wide analysis of RNA structures (Table 1) [91]. This method involves specific enzyme (RNase V1 and S1) treatments and deep sequencing of RNA fragments [92]. In contrast with other methods, structural data from 3000 transcripts can be obtained in a single experiment [91]. By analyzing mRNA structures, Kertesz *et al.* found that coding regions were much more structured than the untranslated regions (UTRs), suggesting that the less structured UTRs may expose functional elements, while the highly structured coding regions tend to be protected from conformational changes and have the potential to regulate ribosome translocation [91]. Additionally, PARS has been applied for the investigations of riboSNitches, which are important RNA elements strongly related to structural changes [93]. Even though PARS has not been commonly applied in investigations of the functional domains of lncRNAs, Ilik *et al.* demonstrated the accuracy of this method by comparing the datasets to the results obtained by SHAPE, and their results were concordant [34]. PARS is the first high-throughput approach for the genome-wide elucidation of RNA structural properties [94], and it will undoubtedly play a significant role in further structural analyses of lncRNA.

Another nuclease-based approach is fragmentation sequencing (FragSeq), which utilizes P1 endonuclease to digest single-stranded RNA, followed by high-throughput sequencing and bioinformatic analyses of the generated fragments (Table 1) [95]. Although only single-stranded RNA regions can be directly identified using this approach, its biggest advantage lies in endogenous control, which shows the ability to recognize 5’ phosphate and 5’ hydroxyl residues that are not generated by nuclease digestion, significantly increasing the accuracy of this method [94]. The feasibility and reproducibility of this method have been validated by the identification of the entire mouse nuclear transcriptome, leading to the discovery of novel conserved structures of ncRNAs [95].

#### 3.2.3. ss/dsRNA-seq Techniques

ss/dsRNA-seq methods were the first high throughput nuclease-based approaches used for the investigations of RNA structures in plants (Table 1) [90]. ssRNA-seq and dsRNA-seq, using RNase I (an ssRNase) and RNase V1 (a dsRNase), respectively, can specifically differentiate between single-stranded RNAs and base-paired RNAs. In contrast to the site-specific cleavage in PARS and FragSeq, all the ssRNA or dsRNA in a sample is digested by nuclease, offering greater sequencing depth and providing better structural information [96]. For instance, Zheng *et al.* determined the functional significance of base-paired RNAs in *A. thaliana* using dsRNA-seq. They found that the exons of *A. thaliana* genome enriched with many base pairings were significantly less evolutionarily conserved than other regions, such as 3’UTRs, 5’UTRs, and introns, suggesting that base-pairing interactions were disfavored in the protein-coding regions of plant mRNAs [97]. In addition, dsRNA-seq has been applied to interrogate the dsRNA component of the *A. thaliana* transcriptome. Through combining dsRNA-seq with smRNA-seq, they identified ~200 new smRNA-producing substrates of RDR6 (RNA-dependent RNA polymerase 6) [97]. Even though either method can be performed in order to investigate the RNA structure, their use in combination can further increase the probing accuracy [16]. To date, ss/dsRNA-seq remain robust approaches for the investigation of RNA structures, and have been successfully applied for the determination of RNA structure–function relationships in *A. thaliana*, *Drosophila*, and *Caenorhabditis elegans* [97,98,99].

### 3.3. RNA Structure Probing in Vivo

#### 3.3.1. DMS-seq, Structural-seq, and Mod-seq

RNA structure probing *in vitro* can provide information about RNA secondary structure, but *in vitro* results cannot be completely extrapolated to *in vivo* conditions. Therefore, *in vivo* methods for RNA structure probing are urgently needed in order to decode RNA structure–function relationships. Currently, different chemical reagents that are able to penetrate rapidly into all cellular compartments are used in these *in vivo* methods. DMS, which directly methylates the base-paring faces of A and C of RNA in loops, bugles, mismatches, and joining regions, is the first RNA structure probing reagent used in living cells [100]. In different chemical environments, nucleotides show different DMS reactivities [100]. For example, nucleotides involved in hydrogen bonding show reduced DMS reactivity, while nucleotides in some unusual chemical environments may show higher reactivities [101]. Therefore, the nucleotide chemical environment can be elucidated based on the efficacy of methylation. Three approaches, termed DMS–seq, Structural-seq, and Mod-seq, were designed based on DMS probing methodology (Table 1) [16]. These methodologies differ in terms of the processing steps following the application of DMS. Specifically, the addition of NGS adapters is required on each side of the DMS-modified RNAs for cDNA generation in Mod-seq [102,103]. In DMS-seq, only 3’ NGS adapters are fused to the fragmented RNAs, while Structure-seq involves random hexamer (N_6_) reverse transcription for the first strand cDNA synthesis and the addition of a part of NGS adapter on one side. Additionally, cDNA ligation differs between all three methods. Structural-seq uses linear DNA ligation, while intramolecular circular DNA ligation is used in DMS-seq and Mod-seq. Furthermore, DMS-seq and Structural-seq are used for the investigations of polyadenylated transcripts, while Mod-seq can be used to study total RNA [16].

These techniques have been used to determine the secondary structures of coding and non-coding RNAs. Rouskin *et al.* used DMS-seq to probe mRNA structures in yeast and mammalian cells, showing an excellent agreement with the previously determined mRNA structures [104]. Ding *et al.* investigated RNA structures of *A. thaliana* *in vivo* by Structure-seq, and found a three-nucleotide periodic repeat pattern in the coding regions, which was closely associated with translational efficiency [105]. The structural information of four rRNAs and 32 additional RNAs in yeast was determined by Mod-seq. Furthermore, Mod-seq has been proven to be a robust method for the investigations of the structures of long RNAs and complex RNA mixtures, because of its correct detection of structural changes in 5.8S and 25S rRNAs in the ribosomal protein L26 deletion mutant [102]. Although these methods have been widely used in RNA structural studies, several disadvantages remain. For example, DMS reagent has a limited shelf life, and the use of a reagent that is not fresh can lead to poor target modification and high error rates [100]. The selection of primers should be carefully considered, because the use of primers with poor specificity and labeling efficiency can result in multiple unwanted disruptions of the process [100]. Furthermore, the ability of DMS to differentiate between dsRNA and ssRNA is hindered when ssRNA interacts with RNA binding protein (RBP) *in vivo* [106]. Therefore, a more suitable chemical reagent needs to be developed in order for these issues to be resolved.

#### 3.3.2. icSHAPE

A traditional SHAPE reagent can be used for highly accurate studies of RNA structures composed of all four nucleotides [107]. However, the high background signal obtained by the traditional SHAPE probing methods increases false positive rates. Additionally, RNA structural information obtained *in vitro* greatly differs from its dynamic structure *in vivo*. In contrast to this, DMS allows for RNA structure probing *in vivo*, but only two of the four nucleotides can be modified, which often leads to incorrect results [107]. Because of this, a new method termed *In vivo* Click SHAPE (icSHAPE), using an improved SHAPE reagent for genome-wide investigations of RNA structure, has been created (Table 1) [108]. The existing SHAPE probe 2-methylnicotinic acid imidazolide (NAI) is changed into NAI-N_3_ by adding an azide group, making it possible for RNA structure probing *in vivo* [108]. This azide group plays a very important role in the subsequent “click” of biotin moiety to SHAPE reagent, which allows for the purification of NAI-N_3_-modified RNA through streptavidin beads, and the signal to noise ratio of sequencing results vastly increases after the enrichment of modified RNAs [108]. The accuracy and reproducibility of icSHAPE have been validated by studying the known structures of 18S and 28S rRNAs in mouse embryonic stem cells (mESC) [107]. Furthermore, icSHAPE showed that 3’ UTR structures tend to be more single-stranded than CDS or 5’ UTR. ncRNAs, such as pseudogenes, lncRNAs, and primary miRNA precursors, tend to be more folded *in vivo*, suggesting that mRNA and ncRNA structures differ greatly *in vivo* [108].

#### 3.3.3. CLASH and hiCLIP

Most of the RNA structure probing methods, such as DMS-seq, SHAPE-seq, PARS, and FragSeq, can only determine the individual base content in secondary structure, while the information about paired regions involved in higher order structure remains unknown, which prevents the rapid decoding of RNA higher order structures. Crosslinking Ligation and Sequencing Hybrids (CLASH) method, designed by Tollervey’s lab, has been successfully used for the studies of intermolecular or intramolecular RNA–RNA interactions as well as the functional structures formed by paired regions (Table 1) [109]. The sensitivity and accuracy of this method were assessed by the identification of the known target sites for box C/D modification-guide snoRNA in yeast. The results were shown to be in agreement with the previous ones [109]. Additionally, multiple base paired regions between U3 snoRNA and pre-rRNA strongly facilitate pre-rRNA folding and its subsequent processing, suggesting the significant contribution of intramolecular interactions to the maintenance of RNA secondary structure [110]. CLASH was applied for the mapping of the human interactome, and Helwak *et al.* found that majority of miRNAs interact with mRNAs through 5’ seed region [109]. Furthermore, nearly 60% of miRNA-mRNA interactions are achieved by non-canonical base pairing, containing bulges, loops, and hairpins, which may affect the response of RNA-induced silencing complex (RISC) to miRNA-target binding [109].

Another probing method, with a similar approach to the previous one, is hiCLIP (RNA hybrid and individual-nucleotide resolution UV cross-linking and immunoprecipitation) (Table 1) [111]. Compared with CLASH, hiCLIP shows a greater control over the ligation of two RNA strands. Sugimoto *et al.* applied hiCLIP in the studies of duplex structures bound by a dsRBP, termed Staufen 1 (STAU1), which is involved in mRNA localization, stability, and translation. The results showed that almost 70% of duplexes can be found in 3’ UTR and duplexes in CDS tend to have shorter loops than in the UTRs [111]. In addition, hiCLIP identified an 858-nt-long duplex region in the 3’ UTR of XBP1, a STAU1 negatively-regulated mRNA. This duplex was found to play a central role in the regulation of XBP1 stability. A decrease in this stability was observed when the structure of the duplex was disrupted by AA dinucleotide insertion, while its stability returned to the original levels when a complementary TT dinucleotide was inserted, demonstrating a close structure–function relationships [111]. Nevertheless, icCLIP shows severe limitations in the probing of other RNA secondary structures that are not involved in RBP interactions.

#### 3.3.4. RNA Proximity Ligation (RPL)

Ramani *et al.* developed a more general method, based on the principles similar to the principles of CLASH and hiCLIP, called RPL (RNA Proximity Ligation) (Table 1) [112]. In contrast to the cDNA library construction in chemical probing methods, RPL library is generated by *in situ* RNase digestion of RNA and treatment with exogenous T4 RNA Ligase I. This is followed by high-throughput sequencing, using these chimeric molecules formed by RNA ligation. The pairwise data can be obtained by analyzing chimeric reads [112]. RPL generates the pairwise data of rRNA and other abundant RNAs, such as snoRNA (*snR86*), U1 spliceosome RNA (*snR19*), and U2 spliceosomal RNA homolog (*LSR1*) in yeast and human cells [112]. The well-characterized interacting regions show high RPL scores, demonstrating its superior accuracy and reproducibility. However, this method requires further improvements in orderfor its accuracy and range of application to be increased. The following modifications are needed: First, since a high rate of background noise is always obtained for promiscuous ligation events, enzymatic protocols for RNA purification should be optimized, in order to increase the abundance of the investigated RNA molecules [106]. Additionally, RPL can provide 2D RNA structural models, but these data are often lower-resolution, while conventional RNA structure probing methods, such as DMS-seq and SHAPE-seq, even though they are able to provide higher-resolution data, generate only 1D RNA structural models. Therefore, combining the advantages of RPL and conventional probing methods may be very beneficial for future research [112]. Nevertheless, RPL has initiated the studies of RNA structures from a different angle, providing new mechanistic insights into pairwise interactions within RNA secondary structures.

## 4. Conclusions and Future Direction

lncRNAs play significant roles during transcription, post-transcription, and epigenetic processes in living cells [10,113,114]. Recently, a large number of lncRNAs have been discovered, butvery few of their molecular mechanisms have been characterized, leaving their structure–function relationships undefined [19]. Even though RNA structure probing methods have been developing rapidly, most of them are able to obtain only secondary structure data, which sometimes cannot sufficiently explain structure–function relationships. More detailed information about the tertiary structures of RNAs is required [115]. Furthermore, each of these methods has its disadvantages, although they can be used for the determination of the functional sites in RNAs [80,100]. For example, nucleotides that are not involved in Watson–Crick base pairing but are involved in non-canonical interactions are apparently protected from SHAPE reactions, while DMS is able to react with these nucleotides [100]. Therefore, the complementary usage of different methods is indispensable for an accurate and comprehensive understanding of lncRNA structures. Furthermore, most of the identified lncRNA structures were determined *in vitro*, but lncRNA structures *in vivo*, which are less structured and more dynamic, can often differ dramatically from the *in vitro* structures. One possible reason for this is that some reagents lack the ability to penetrate cells, which severely limits their usage *in vivo* [86,100,105]. More importantly, lncRNAs can interact with proteins, DNAs, and other RNAs *in vivo*, which may inhibit or affect the interactions between these reagents and target lncRNAs. Therefore, the development of new methods that can solve the currently existing problems is urgently required. An increasing number of identified lncRNA conserved structures will provide an improved understanding of lncRNA biological functions.

## Figures and Tables

**Table 1:** Summary of RNA structural probing methods.

Methods	Application to Date	Probe	Data	References
SHAPE-seq	*In vitro*	1M7, NMIA	1D	[87]
SHAPE-MAP	*In vitro*	1M7	1D	[88]
RING-Map	*In vitro*	DMS	1D	[89]
PARS	*In vitro*	RNase S1 (ssRNA); RNase V1 (dsRNA)	1D	[91]
Fragseq	*In vitro*	RNase P1 (ssRNA)	1D	[95]
ss/dsRNA-seq	*In vitro*	RNase I (ssRNA); RNase V1 (dsRNA)	1D	[90,97]
Mod-seq	*In vivo*	DMS	1D	[102,103]
DMS-seq	*In vivo*	DMS	1D	[104]
Structural-seq	*In vivo*	DMS	1D	[105]
icSHAPE	*In vivo*	NAI-N_3_	1D	[107,108]
CLASH	*In vivo*	UV crosslinking	2D	[109,110]
hiCLIP	*In vivo*	UV crosslinking	2D	[111]
RPL	*In vivo*	No crosslinking	2D	[112]

## Abbreviations

ncRNA: non-coding RNA; snRNA: small nuclear RNA; snoRNA: small nucleolar RNA; SRA: steroid receptor RNA activator; NMR: nuclear magnetic resonance; XCI: X-chromosome inactivation; PRC2: polycomb repressive 2; H3K27me3: histone H3 lysine K27 trimethylation CRISPR/Cas9: Clustered regularly interspaced short palindromic repeats (CRISPR)-associated endonuclease 9; roX1: RNA on the X 1; roX2: RNA on the X 2; HAS: high-affinity sites; H4K16ac: histone H4 on lysine16 acetylation; MSL: Male-lethal specific; MLE: maleless; MSL2: male-specific lethal 2 homolog; LSD1: lysine-specific demethylase 1; HOTAIR:HOX antisense intergenic RNA; minHOTAIR: minimal binding motif of HOTAIR; MALAT1: Metastasis associated lung adenocarcinoma transcript 1; mascRNA: MALAT1-associated small cytoplasmic RNA; NEAT2: nuclear enriched abundant transcript 2; Gas5: growth arrest-specific 5; GR: glucocorticoid receptor; GC: glucocorticoid; IPS1: Induced by Phosphate Starvation 1; eTM: endogenous target mimicry mechanism; FLC: Flowering locus C; COOLAIR: Cold Induced Long Antisense Intergenic noncoding RNA; COLDAIR: Cold Assisted Intronic noncoding RNA; LDMAR: long-day-specific male-fertility-associated lincRNA; PSMS: photoperiod-sensitive male sterility; NK 58S: Nongken 58S; NK 58N: Nongken 58N; RdDM: RNA-dependent DNA methylation; RBP: RNA binding proteins; MtRBP1: Medicago truncatula RNA Binding Protein 1; DMS: dimethyl sulfate; SHAPE: selective 2’-hydroxyl acylation analyzed by primer extension; CMCT: 1-cyclohexyl-(2-morpholinoethyl) carbodiimidemetho-p-toluene sulfonate; RNase: ribonuclease; PARS: Parallel analysis of RNA structure; Frag-Seq: fragmentation sequencing; icSHAPE: *In Vivo* Click SHAPE; CLASH : Crosslinking Ligation and Sequencing Hybrids; hiCLIP : RNA hybrid and individual-nucleotide resolution UV cross-linking and immunoprecipitation; RPL : RNA Proximity Ligation.

## References

[1] Djebali S., Davis C.A., Merkel A., Dobin A., Lassmann T., Mortazavi A., Tanzer A., Lagarde J., Lin W., Schlesinger F. (2012). Landscape of transcription in human cells. Nature.

[2] Quinn J.J., Chang H.Y. (2016). Unique features of long non-coding RNA biogenesis and function. Nat. Rev. Genet..

[3] Shabalina S.A., Ogurtsov A.Y., Spiridonov N.A. (2006). A periodic pattern of mRNA secondary structure created by the genetic code. Nucleic Acids Res..

[4] Ponting C.P., Belgard T.G. (2010). Transcribed dark matter: Meaning or myth?. Hum. Mol. Genet..

[5] Kim E.D., Sung S. (2012). Long noncoding RNA: Unveiling hidden layer of gene regulatory networks. Trends Plant Sci..

[6] Simon S.A., Meyers B.C. (2011). Small RNA-mediated epigenetic modifications in plants. Curr. Opin. Plant Biol..

[7] Yang Y., Wen L., Zhu H. (2015). Unveiling the hidden function of long non-coding RNA by identifying its major partner-protein. Cell Biosci..

[8] Chen L.L., Carmichael G.G. (2010). Decoding the function of nuclear long non-coding RNAs. Curr. Opin. Cell Biol..

[9] Wang K.C., Chang H.Y. (2011). Molecular mechanisms of long noncoding RNAs. Mol. Cell.

[10] Mercer T.R., Mattick J.S. (2013). Structure and function of long noncoding RNAs in epigenetic regulation. Nat. Struct. Mol. Biol..

[11] Johnsson P., Lipovich L., Grander D., Morris K.V. (2014). Evolutionary conservation of long non-coding RNAs; sequence, structure, function. Biochim. Biophys. Acta.

[12] Novikova I.V., Hennelly S.P., Sanbonmatsu K.Y. (2012). Structural architecture of the human long non-coding RNA, steroid receptor RNA activator. Nucleic Acids Res..

[13] Zhang H., Chen X., Wang C., Xu Z., Wang Y., Liu X., Kang Z., Ji W. (2013). Long non-coding genes implicated in response to stripe rust pathogen stress in wheat (*Triticum aestivum* L.). Mol. Biol. Rep..

[14] Di C., Yuan J., Wu Y., Li J., Lin H., Hu L., Zhang T., Qi Y., Gerstein M.B., Guo Y. (2014). Characterization of stress-responsive lncRNAs in *Arabidopsis thaliana* by integrating expression, epigenetic and structural features. Plant J..

[15] Dominguez C., Schubert M., Duss O., Ravindranathan S., Allain F.H. (2011). Structure determination and dynamics of protein-RNA complexes by NMR spectroscopy. Prog. Nucl. Magn. Reson. Spectrosc..

[16] Kwok C.K., Tang Y., Assmann S.M., Bevilacqua P.C. (2015). The RNA structurome: Transcriptome-wide structure probing with next-generation sequencing. Trends Biochem. Sci..

[17] Guttman M., Amit I., Garber M., French C., Lin M.F., Feldser D., Huarte M., Zuk O., Carey B.W., Cassady J.P. (2009). Chromatin signature reveals over a thousand highly conserved large non-coding RNAs in mammals. Nature.

[18] Novikova I.V., Hennelly S.P., Tung C.S., Sanbonmatsu K.Y. (2013). Rise of the RNA machines: Exploring the structure of long non-coding RNAs. J. Mol. Biol..

[19] Derrien T., Johnson R., Bussotti G., Tanzer A., Djebali S., Tilgner H., Guernec G., Martin D., Merkel A., Knowles D.G. (2012). The gencode v7 catalog of human long noncoding RNAs: Analysis of their gene structure, evolution, and expression. Genome Res..

[20] Simon M.D., Pinter S.F., Fang R., Sarma K., Rutenberg-Schoenberg M., Bowman S.K., Kesner B.A., Maier V.K., Kingston R.E., Lee J.T. (2013). High-resolution Xist binding maps reveal two-step spreading during X-chromosome inactivation. Nature.

[21] Pontier D.B., Gribnau J. (2011). Xist regulation and function explored. Hum. Genet..

[22] Froberg J.E., Yang L., Lee J.T. (2013). Guided by RNAs: X-inactivation as a model for lncRNA function. J. Mol. Biol..

[23] Yang C., Chapman A.G., Kelsey A.D., Minks J., Cotton A.M., Brown C.J. (2011). X-chromosome inactivation: Molecular mechanisms from the human perspective. Hum. Genet..

[24] Morey C., Arnaud D., Avner P., Clerc P. (2001). Tsix-mediated repression of Xist accumulation is not sufficent for normal random X inactivation. Hum. Mol. Genet..

[25] Migeon B.R., Lee C.H., Chowdhury A.K., Carpenter H. (2002). Species differences in Tsix/Tsix reveal the roles of these genes in X-chromosome inactivation. Am. J. Hum. Genet..

[26] Maenner S., Blaud M., Fouillen L., Savoye A., Marchand V., Dubois A., Sanglier-Cianferani S., van Dorsselaer A., Clerc P., Avner P. (2010). 2-D structure of the a region of Xist RNA and its implication for PRC2 association. PLoS Biol..

[27] Duszczyk M.M., Wutz A., Rybin V., Sattler M. (2011). The Xist RNA A-repeat comprises a novel AUCG tetraloop fold and a platform for multimerization. RNA.

[28] Jeon Y., Lee J.T. (2011). Yy1 tethers Xist RNA to the inactive X nucleation center. Cell.

[29] Fang R., Moss W.N., Rutenberg-Schoenberg M., Simon M.D. (2015). Probing Xist RNA structure in cells using targeted structure-seq. PLoS Genet..

[30] Lv Q., Yuan L., Song Y., Sui T., Li Z., Lai L. (2016). D-repeat in the *Xist* gene is required for X chromosome inactivation. RNA Biol..

[31] Flintoft L. (2013). Non-coding RNA: Structure and function for lncRNAs. Nat. Rev. Genet..

[32] Wutz A. (2013). Noncoding RoX RNA remodeling triggers fly dosage compensation complex assembly. Mol. Cell.

[33] Maenner S., Muller M., Frohlich J., Langer D., Becker P.B. (2013). ATP-dependent RoX RNA remodeling by the helicase maleless enables specific association of MSL proteins. Mol. Cell.

[34] Ilik I.A., Quinn J.J., Georgiev P., Tavares-Cadete F., Maticzka D., Toscano S., Wan Y., Spitale R.C., Luscombe N., Backofen R. (2013). Tandem stem-loops in RoX RNAs act together to mediate X chromosome dosage compensation in drosophila. Mol. Cell.

[35] Gupta R.A., Shah N., Wang K.C., Kim J., Horlings H.M., Wong D.J., Tsai M.C., Hung T., Argani P., Rinn J.L. (2010). Long non-coding RNA HOT AIR reprograms chromatin state to promote cancer metastasis. Nature.

[36] Yan K., Arfat Y., Li D., Zhao F., Chen Z., Yin C., Sun Y., Hu L., Yang T., Qian A. (2016). Structure prediction: New insights into decrypting long noncoding RNAs. Int. J. Mol. Sci..

[37] Tsai M.C., Manor O., Wan Y., Mosammaparast N., Wang J.K., Lan F., Shi Y., Segal E., Chang H.Y. (2010). Long noncoding RNA as modular scaffold of histone modification complexes. Science.

[38] Loewen G., Jayawickramarajah J., Zhuo Y., Shan B. (2014). Functions of lncRNA HOT AIR in lung cancer. J. Hematol. Oncol..

[39] Wang B., Su Y., Yang Q., Lv D., Zhang W., Tang K., Wang H., Zhang R., Liu Y. (2015). Overexpression of long non-coding RNA HOT AIR promotes tumor growth and metastasis in human osteosarcoma. Mol. Cells.

[40] He S., Liu S., Zhu H. (2011). The sequence, structure and evolutionary features of HOTAIR in mammals. BMC Evol. Biol..

[41] Wu L., Murat P., Matak-Vinkovic D., Murrell A., Balasubramanian S. (2013). Binding interactions between long noncoding RNAHOTAIR and PRC2 proteins. Biochemistry.

[42] Somarowthu S., Legiewicz M., Chillon I., Marcia M., Liu F., Pyle A.M. (2015). HOTAIR forms an intricate and modular secondary structure. Mol. Cell.

[43] Yoshimoto R., Mayeda A., Yoshida M., Nakagawa S. (2016). MALAT1 long non-coding RNA in cancer. Biochim. Biophys. Acta.

[44] West J.A., Davis C.P., Sunwoo H., Simon M.D., Sadreyev R.I., Wang P.I., Tolstorukov M.Y., Kingston R.E. (2014). The long noncoding RNAs NEAT1 and MALAT1 bind active chromatin sites. Mol. Cell.

[45] Zhang J., Zhang B., Wang T., Wang H. (2015). LncRNA malat1 overexpression is an unfavorable prognostic factor in human cancer: Evidence from a meta-analysis. Int. J. Clin. Exp. Med..

[46] Tripathi V., Ellis J.D., Shen Z., Song D.Y., Pan Q., Watt A.T., Freier S.M., Bennett C.F., Sharma A., Bubulya P.A. (2010). The nuclear-retained noncoding RNA MALAT1 regulates alternative splicing by modulating Sr splicing factor phosphorylation. Mol. Cell.

[47] Wilusz J.E., Freier S.M., Spector D.L. (2008). 3ʹ end processing of a long nuclear-retained noncoding RNA yields a tRNA-like cytoplasmic RNA. Cell.

[48] Wilusz J.E., JnBaptiste C.K., Lu L.Y., Kuhn C.D., Joshua-Tor L., Sharp P.A. (2012). A triple helix stabilizes the 3ʹ ends of long noncoding RNAs that lack poly(A) tails. Genes Dev..

[49] Brown J.A., Valenstein M.L., Yario T.A., Tycowski K.T., Steitz J.A. (2012). Formation of triple-helical structures by the 3’-endsequences of MALAT1 and MENβ noncoding RNAs. Proc. Natl. Acad. Sci. USA.

[50] Brown J.A., Bulkley D., Wang J., Valenstein M.L., Yario T.A., Steitz T.A., Steitz J.A. (2014). Structural insights into the stabilization of MALAT1 noncoding RNA by a bipartite triple helix. Nat. Struct. Mol. Biol..

[51] Pickard M.R., Williams G.T. (2015). Molecular and cellular mechanisms of action of tumour suppressor Gas5 lncRNA. Genes (Basel).

[52] Kino T., Hurt D.E., Ichijo T., Nader N., Chrousos G.P. (2010). Noncoding RNAGas5 is a growth arrest- and starvation-associated repressor of the glucocorticoid receptor. Sci. Signal..

[53] Rinn J.L., Chang H.Y. (2012). Genome regulation by long noncoding RNAs. Annu. Rev. Biochem..

[54] Liu J., Wang H., Chua N.H. (2015). Long noncoding RNA transcriptome of plants. Plant Biotechnol. J..

[55] Li X., Wu Z., Fu X., Han W. (2014). LncRNAs: Insights into their function and mechanics in underlying disorders. Mutat. Res. Rev. Mutat. Res..

[56] Wierzbicki A.T. (2012). The role of long non-coding RNA in transcriptional gene silencing. Curr. Opin. Plant Biol..

[57] Zhang Y.C., Chen Y.Q. (2013). Long noncoding RNAs: New regulators in plant development. Biochem. Biophys. Res. Commun..

[58] Franco-Zorrilla J.M., Valli A., Todesco M., Mateos I., Puga M.I., Rubio-Somoza I., Leyva A., Weigel D., Garcia J.A., Paz-Ares J. (2007). Target mimicry provides a new mechanism for regulation of microRNA activity. Nat. Genet..

[59] Heo J.B., Lee Y.S., Sung S. (2013). Epigenetic regulation by long noncoding RNAs in plants. Chromosome Res..

[60] Guil S., Esteller M. (2015). RNA–RNA interactions in gene regulation: The coding and noncoding players. Trends Biochem. Sci..

[61] Yamaguchi A., Abe M. (2012). Regulation of reproductive development by non-coding RNA in *Arabidopsis*: To flower or not to flower. J. Plant Res..

[62] Csorba T., Questa J.I., Sun Q., Dean C. (2014). Antisense coolair mediates the coordinated switching of chromatin states at FLC during vernalization. Proc. Natl. Acad. Sci. USA.

[63] Kim D.H., Sung S. (2012). Environmentally coordinated epigenetic silencing of FLC by protein and long noncoding RNA components. Curr. Opin. Plant Biol..

[64] Wang Z.W., Wu Z., Raitskin O., Sun Q., Dean C. (2014). Antisense-mediated FLC transcriptional repression requires the P-TEFb transcription elongation factor. Proc. Natl. Acad. Sci. USA.

[65] Sun Q., Csorba T., Skourti-Stathaki K., Proudfoot N.J., Dean C. (2013). R-loop stabilization represses antisense transcription at the *Arabidopsis* FLC locus. Science.

[66] Chekanova J.A. (2015). Long non-coding RNAs and their functions in plants. Curr. Opin. Plant Biol..

[67] Lee J.T. (2009). Lessons from X-chromosome inactivation: Long ncRNA as guides and tethers to the epigenome. Genes Dev..

[68] Zhao J., Ohsumi T.K., Kung J.T., Ogawa Y., Grau D.J., Sarma K., Song J.J., Kingston R.E., Borowsky M., Lee J.T. (2010). Genome-wide identification of polycomb-associated RNAs by RIP-seq. Mol. Cell.

[69] Ding J., Lu Q., Ouyang Y., Mao H., Zhang P., Yao J., Xu C., Li X., Xiao J., Zhang Q. (2012). A long noncoding RNA regulates photoperiod-sensitive male sterility, an essential component of hybrid rice. Proc. Natl. Acad. Sci. USA.

[70] Ding J., Shen J., Mao H., Xie W., Li X., Zhang Q. (2012). RNA-directed DNA methylation is involved in regulating photoperiod-sensitive male sterility in rice. Mol. Plant.

[71] Zhang J., Mujahid H., Hou Y., Nallamilli B.R., Peng Z. (2013). Plant long ncRNAs: A new frontier for gene regulatory control. Am. J. Plant Sci..

[72] Bardou F., Merchan F., Ariel F., Crespi M. (2011). Dual RNAs in plants. Biochimie.

[73] Gultyaev A.P., Roussis A. (2007). Identification of conserved secondary structures and expansion segments in ENOD40 RNAs reveals new ENOD40 homologues in plants. Nucleic Acids Res..

[74] Ariel F., Romero-Barrios N., Jegu T., Benhamed M., Crespi M. (2015). Battles and hijacks: Noncoding transcription in plants. Trends Plant Sci..

[75] Rohrig H., Schmidt J., Miklashevichs E., Schell J., John M. (2002). Soybean ENOD40 encodes two peptides that bind to sucrose synthase. Proc. Natl. Acad. Sci. USA.

[76] Girard G., Roussis A., Gultyaev A.P., Pleij C.W., Spaink H.P. (2003). Structural motifs in the RNA encoded by the early nodulation gene enod40 of soybean. Nucleic Acids Res..

[77] Campalans A., Kondorosi A., Crespi M. (2004). Enod40, a short open reading frame-containing mRNA, induces cytoplasmic localization of a nuclear RNA binding protein in medicago truncatula. Plant Cell.

[78] Anderson D.M., Anderson K.M., Chang C.L., Makarewich C.A., Nelson B.R., McAnally J.R., Kasaragod P., Shelton J.M., Liou J., Bassel-Duby R. (2015). A micropeptide encoded by a putative long noncoding RNA regulates muscle performance. Cell.

[79] Bardou F., Ariel F., Simpson C.G., Romero-Barrios N., Laporte P., Balzergue S., Brown J.W., Crespi M. (2014). Long noncoding RNA modulates alternative splicing regulators in *Arabidopsis*. Dev. Cell.

[80] Ziehler W.A., Engelke D.R. (2001). Probing RNA structure with chemical reagents and enzymes. Curr. Protoc. Nucleic Acid Chem..

[81] Cheong H.K., Hwang E., Lee C., Choi B.S., Cheong C. (2004). Rapid preparation of RNA samples for NMR spectroscopy and X-ray crystallography. Nucleic Acids Res..

[82] Batey R.T., Kieft J.S. (2007). Improved native affinity purification of RNA. RNA.

[83] Said N., Rieder R., Hurwitz R., Deckert J., Urlaub H., Vogel J. (2009). *In vivo* expression and purification of aptamer-tagged small RNA regulators. Nucleic Acids Res..

[84] Chillon I., Marcia M., Legiewicz M., Liu F., Somarowthu S., Pyle A.M. (2015). Native purification and analysis of long RNAs. Methods Enzymol..

[85] Poulsen L.D., Kielpinski L.J., Salama S.R., Krogh A., Vinther J. (2015). SHAPE selection (SHAPES) enrich for RNA structure signal in SHAPE sequencing-based probing data. RNA.

[86] Spitale R.C., Crisalli P., Flynn R.A., Torre E.A., Kool E.T., Chang H.Y. (2013). RNA SHAPE analysis in living cells. Nat. Chem. Biol..

[87] Lucks J.B., Mortimer S.A., Trapnell C., Luo S., Aviran S., Schroth G.P., Pachter L., Doudna J.A., Arkin A.P. (2011). Multiplexed RNA structure characterization with selective 2′-hydroxyl acylation analyzed by primer extension sequencing (SHAPE-seq). Proc. Natl. Acad. Sci. USA.

[88] Siegfried N.A., Busan S., Rice G.M., Nelson J.A., Weeks K.M. (2014). RNA motif discovery by shape and mutational profiling (SHAPE-MAP). Nat. Methods.

[89] Homan P.J., Favorov O.V., Lavender C.A., Kursun O., Ge X., Busan S., Dokholyan N.V., Weeks K.M. (2014). Single-molecule correlated chemical probing of RNA. Proc. Natl. Acad. Sci. USA.

[90] Foley S.W., Vandivier L.E., Kuksa P.P., Gregory B.D. (2015). Transcriptome-wide measurement of plant RNA secondary structure. Curr. Opin. Plant Biol..

[91] Kertesz M., Wan Y., Mazor E., Rinn J.L., Nutter R.C., Chang H.Y., Segal E. (2010). Genome-wide measurement of RNA secondary structure in yeast. Nature.

[92] Wan Y., Qu K., Ouyang Z., Chang H.Y. (2013). Genome-wide mapping of RNA structure using nuclease digestion and high-throughput sequencing. Nat. Protoc..

[93] Wan Y., Qu K., Zhang Q.C., Flynn R.A., Manor O., Ouyang Z., Zhang J., Spitale R.C., Snyder M.P., Segal E. (2014). Landscape and variation of RNA secondary structure across the human transcriptome. Nature.

[94] Novikova I.V., Hennelly S.P., Sanbonmatsu K.Y. (2013). Tackling structures of long noncoding RNAs. Int. J. Mol. Sci..

[95] Underwood J.G., Uzilov A.V., Katzman S., Onodera C.S., Mainzer J.E., Mathews D.H., Lowe T.M., Salama S.R., Haussler D. (2010). Fragseq: Transcriptome-wide RNA structure probing using high-throughput sequencing. Nat. Methods.

[96] Kashi K., Henderson L., Bonetti A., Carninci P. (2016). Discovery and functional analysis of lncRNAs: Methodologies to investigate an uncharacterized transcriptome. Biochim. Biophys. Acta.

[97] Zheng Q., Ryvkin P., Li F., Dragomir I., Valladares O., Yang J., Cao K., Wang L.S., Gregory B.D. (2010). Genome-wide double-stranded RNA sequencing reveals the functional significance of base-paired RNAs in *Arabidopsis*. PLoS Genet..

[98] Li F., Zheng Q., Ryvkin P., Dragomir I., Desai Y., Aiyer S., Valladares O., Yang J., Bambina S., Sabin L.R. (2012). Global analysis of RNA secondary structure in two metazoans. Cell Rep..

[99] Li F., Zheng Q., Vandivier L.E., Willmann M.R., Chen Y., Gregory B.D. (2012). Regulatory impact of RNA secondary structure across the *Arabidopsis* transcriptome. Plant Cell.

[100] Cordero P., Kladwang W., VanLang C.C., Das R. (2012). Quantitative dimethyl sulfate mapping for automated RNA secondary structure inference. Biochemistry.

[101] Kubota M., Tran C., Spitale R.C. (2015). Progress and challenges for chemical probing of RNA structure inside living cells. Nat. Chem. Biol..

[102] Talkish J., May G., Lin Y., Woolford J.L., McManus C.J. (2014). Mod-seq: High-throughput sequencing for chemical probing of RNA structure. RNA.

[103] Lin Y., May G.E., Joel McManus C. (2015). Mod-seq: A high-throughput method for probing RNA secondary structure. Methods Enzymol..

[104] Rouskin S., Zubradt M., Washietl S., Kellis M., Weissman J.S. (2014). Genome-wide probing of RNA structure reveals active unfolding of mRNA structures *in vivo*. Nature.

[105] Ding Y., Tang Y., Kwok C.K., Zhang Y., Bevilacqua P.C., Assmann S.M. (2014). *In vivo* genome-wide profiling of RNA secondary structure reveals novel regulatory features. Nature.

[106] Lu Z., Chang H.Y. (2016). Decoding the RNA structurome. Curr. Opin. Struct. Biol..

[107] Flynn R.A., Zhang Q.C., Spitale R.C., Lee B., Mumbach M.R., Chang H.Y. (2016). Transcriptome-wide interrogation of RNA secondary structure in living cells with icSHAPE. Nat. Protoc..

[108] Spitale R.C., Flynn R.A., Zhang Q.C., Crisalli P., Lee B., Jung J.W., Kuchelmeister H.Y., Batista P.J., Torre E.A., Kool E.T. (2015). Structural imprints *in vivo* decode RNA regulatory mechanisms. Nature.

[109] Helwak A., Kudla G., Dudnakova T., Tollervey D. (2013). Mapping the human miRNA interactome by clash reveals frequent noncanonical binding. Cell.

[110] Kudla G., Granneman S., Hahn D., Beggs J.D., Tollervey D. (2011). Cross-linking, ligation, and sequencing of hybrids reveals RNA–RNA interactions in yeast. Proc. Natl. Acad. Sci. USA.

[111] Sugimoto Y., Vigilante A., Darbo E., Zirra A., Militti C., D′Ambrogio A., Luscombe N.M., Ule J. (2015). Hiclip reveals the *in vivo* atlas of mRNA secondary structures recognized by Staufen 1. Nature.

[112] Ramani V., Qiu R., Shendure J. (2015). High-throughput determination of RNA structure by proximity ligation. Nat. Biotechnol..

[113] Cao J. (2014). The functional role of long non-coding RNAs and epigenetics. Biol. Proced. Online.

[114] Yoon J.H., Abdelmohsen K., Gorospe M. (2013). Posttranscriptional gene regulation by long noncoding RNA. J. Mol. Biol..

[115] Novikova I.V., Hennelly S.P., Sanbonmatsu K.Y. (2012). Sizing up long non-coding RNAs do lncRNAs have secondary and tertiary structure. Bioarchitecture.

